# A Possible Link between Supra-Second Open-Ended Timing Sensitivity and Obsessive-Compulsive Tendencies

**DOI:** 10.3389/fnbeh.2016.00127

**Published:** 2016-06-27

**Authors:** Sharon Gilaie-Dotan, Hamutal Ashkenazi, Reuven Dar

**Affiliations:** ^1^UCL Institute of Cognitive Neuroscience, University College LondonLondon, UK; ^2^School of Psychological Sciences, Tel Aviv UniversityTel Aviv, Israel

**Keywords:** obsessive-compulsive disorder, OCD, time perception, supra-second, sub-second

## Abstract

One of the main characteristics of obsessive-compulsive disorder (OCD) is the persistent feeling of uncertainty, affecting many domains of actions and feelings. It was recently hypothesized that OCD uncertainty is related to attenuated access to internal states. As supra-second timing is associated with bodily and interoceptive awareness, we examined whether supra-second timing would be associated with OC tendencies. We measured supra-second (~9 s) and sub-second (~450 ms) timing along with control non-temporal perceptual tasks in a group of 60 university students. Supra-second timing was measured either with fixed criterion tasks requiring to temporally discriminate between two predefined fixed interval durations (9 vs. 9.9 s), or with an open-ended task requiring to discriminate between 9 s and longer intervals which were of varying durations that were not *a priori* known to the participants. The open-ended task employed an adaptive Bayesian procedure that efficiently estimated the duration difference required to discriminate 9 s from longer intervals. We also assessed symptoms of OCD, depression, and anxiety. Open-ended supra-second temporal sensitivity was correlated with OC tendencies, as predicted (even after controlling for depression and anxiety), whereas the other tasks were not. Higher OC tendencies were associated with lower timing sensitivity to 9 s intervals such that participants with higher OC tendency scores required longer interval differences to discriminate 9 s from longer intervals. While these results need to be substantiated in future research, they suggest that open-ended timing tasks, as those encountered in real-life (e.g., estimating how long it would take to complete a task), might be adversely affected in OCD.

## Introduction

A central characteristic of obsessive-compulsive disorder (OCD) is malignant doubt, which affects many domains of actions and feelings and is often followed by a variety of typical pathological behaviors including excessive self-monitoring, repeated checking, mental reconstruction, and repeated demands for external validation or reassurance (Dar, 2004; Nedeljkovic and Kyrios, 2007; American Psychiatric Association, 2013). This persistent doubt has been considered to have a fundamental role in the phenomenology of the disorder (Shapiro, 1965; Tolin et al., 2003; Szechtman and Woody, 2004; American Psychiatric Association, 2013).

A recent model for obsessive-compulsive (OC) doubt and ensuing rituals, termed Seeking Proxies for Internal States (SPIS; Liberman and Dar, 2009; Lazarov et al., 2010), suggests that OC uncertainty can be relevant to any internal state, be it cognitive (e.g., perception, memory, comprehension), affective (e.g., attraction, specific emotions) or bodily (e.g., muscle tension, proprioception) state. Moreover, according to the SPIS hypothesis, doubts concerning one’s internal state are associated with attenuated access to that state. The SPIS model further postulates that OC individuals attempt to compensate for the deficient access to their internal states by developing and relying on *proxies*. Proxies are defined as substitutes for the internal state that the individual perceives as more easily discernible or less ambiguous. Such proxies may be rules, procedures, behaviors or environmental stimuli (Liberman and Dar, 2009). Specifically, according to this model, OC rules, norms and rituals can be viewed as proxies designed to compensate for the reduced access to one’s internal states.

A series of studies that used biofeedback as the external proxy to internal states showed that higher OC tendencies were linked to reduced access to relaxation and muscle tension levels, such that individuals with higher OC tendencies relied more on biofeedback for these internal states. For example, one study showed that subjects with high OC tendencies exhibited reduced performance in judging their own relaxation level and were more reliant on biofeedback to perform the task (Lazarov et al., 2010). Another study showed that higher OC tendencies were associated with reduced accuracy in producing designated muscle tension levels in the absence of biofeedback. Furthermore, participants with high OC tendencies, compared to those with low OC tendencies, were more likely to request the biofeedback even when this involved a potential cost in performance (Lazarov et al., 2012a). Participants with high OC tendencies were also significantly more influenced by false biofeedback when evaluating their own relaxation levels (Lazarov et al., 2010) and muscle tension (Lazarov et al., 2012a) which is consistent with the hypothesized attenuation of these internal states.

The present study was designed to extend these findings by exploring the relationship between OC tendencies and timing abilities. Recent studies show that time perception in the order of seconds (supra-second timing) is linked to interoceptive and bodily awareness (Wittmann et al., 2010; Meissner and Wittmann, 2011). For example, brain activity and brain structure of regions involved in bodily sensation (e.g., insula) were shown to be involved in supra-second time perception (Wittmann et al., 2010; Gilaie-Dotan et al., 2011; Wittmann, 2013 and see Craig, 2009). Furthermore, a recent study suggests that higher interoceptive or bodily awareness (e.g., heartbeat perception) is associated with higher time estimation ability for supra-second durations (Meissner and Wittmann, 2011). This is further supported by studies investigating heart rate variability (HRV) among other autonomic and cardiac measures and their contribution to timing perception (Pollatos et al., 2014b; Cellini et al., 2015). They report that HRV is associated with timing accuracy, such that higher vagal heart control is linked to higher accuracy on timing tasks, and this is true for sub-second (Cellini et al., 2015) or for supra-second timing (Pollatos et al., 2014b). It has also been suggested that the ability to discriminate long supra-second (12 s) durations is linked to mechanisms that maintain self-initiated rhythms, which rely on the neural structures of auditory and somatosensory cortices (Gilaie-Dotan et al., 2011). As supra-second time perception is associated with interoceptive and body awareness, and OC tendency is associated with attenuated access to internal states, we hypothesized that OC tendencies would be negatively correlated with supra-second timing performance. Additional indications point to the possible association between timing and OC tendencies. For example, fronto-striatal circuitry is suggested to support timing and time perception (e.g., Meck, 1996, 2005; Meck and Benson, 2002; Hinton and Meck, 2004) on the one hand, and on the other hand this circuitry is implicated in the pathophysiology of OCD (e.g., Rosenberg and Keshavan, 1998; Maltby et al., 2005; Menzies et al., 2008; Melloni et al., 2012; Milad and Rauch, 2012). In fact many studies show that fronto-striatal activity is modulated in OCD in a variety of tasks as planning, sequence learning, reversal learning, or symptom provocation (Kathmann et al., 2005; Maltby et al., 2005; van den Heuvel et al., 2005; Remijnse et al., 2006; Simon et al., 2010; Freyer et al., 2011). Time perception tasks require participants to use a subjective internal representation of time (“internal clock”) without being able to rely on external clocks providing physical external cues. When these timing tasks involve intervals longer than a few seconds it is assumed that accumulator processes and coincidence detectors are involved (Miall, 1989), to which the fronto-striatal circuits are assumed to contribute [(Meck, 1996, 2005; Meck and Benson, 2002; Hinton and Meck, 2004; Matell and Meck, 2004; Wittmann, 2009; Teki et al., 2011a,b), and see the striatal beat frequency model (Matell and Meck, 2004)]. However, OC symptoms may adversely affect many aspects of the computations that are assumed to be involved in these time perception tasks (e.g., by adding overall noise, diverting attention, increasing stress or emotional states due to the persistent feeling of doubt and uncertainty). While a link between timing and OC tendencies has been suggested by researchers previously (Gu and Kukreja, 2011; Gu et al., 2011), this link has not yet been directly tested in humans.

Timing mechanisms of short durations (typically less than 1–2 s), also termed sub-second timing or “automatic timing”, are supported by different neural and perceptual mechanisms than those supporting timing of longer durations (longer than a few seconds), which are also termed supra-second timing or “cognitive timing” (Elbert et al., 1991; Poppel, 1997; Lewis and Miall, 2003a,b; Ulbrich et al., 2007; Morillon et al., 2009; Gilaie-Dotan et al., 2011). Whereas sub-second timing mechanisms are associated with and rely on motor and sensory processing, supra-second timing involves and relies more on cognitive processes (Lewis and Miall, 2003a). As we hypothesized that OC tendencies would be correlated with supra-second timing performance, and as supra-second timing relies on different mechanisms than sub-second timing, we measured supra-second and sub-second time estimation abilities and correlated both of these with OC tendencies. Specifically, for supra-second timing, we employed a temporal discrimination fixed criterion task requiring to discriminate between two fixed duration intervals (9 vs. 9.9 s), and a temporal sensitivity open-ended task requiring to estimate whether an interval is 9 s or longer. While in the fixed criterion task participants were informed that each trial will involve intervals of either 9 s or almost 10 s, in the open-ended task they were informed that each trial will involve 9 s intervals or longer intervals of varying durations (for further details, see “Materials and Methods” section). Sub-second timing was measured with similar tasks using intervals of ~450 ms or longer duration (540 or 600 ms). We chose larger Weber fractions (of 20 and 33%) for the sub-second paradigms than the fraction used in the supra-second tasks (10%) following pilot testing that indicated that such Weber fractions would yield sub-second accuracy rates comparable to those of the supra-second timing tasks. To examine whether any obtained results were specific to timing, we also administered control (non-temporal) color perception tasks with supra- and sub-second intervals. These control tasks had an identical experimental paradigm apart from requiring participants to discriminate between colors instead of discriminating between intervals (i.e., change of task). These color perception controls tasks were inspired by Coull et al.’s (2004) study, and are assumed to rely on color perception mechanisms that have been shown to dissociate from timing mechanisms both neurally and perceptually (Coull et al., 2004; Gilaie-Dotan et al., 2011, 2014). As OCD is often accompanied by anxiety and depression (American Psychiatric Association, 2013), we also controlled for the levels of these variables using participants’ scores on a self-report scale Depression, Anxiety and Stress Scale (DASS; see “Materials and Methods” section).

## Materials and Methods

### Participants

A group of psychology students (see Table 1 for more details) participated in the study for course credit and provided written informed consent to participate before the study began. The study was approved by the Tel Aviv University human subjects committee. All participants had adequate color vision as verified by Ishihara Color Test (Ishihara, 1917). Inclusion criteria included self-report of normal or corrected-to-normal vision, no history of learning disabilities and no known deficits in sustained attention (a few students were excluded from the study based on self-reports that did not meet these criteria).

**Table 1:** **Descriptive statistics**.

Number of participants	60 (13 men, 47 women)
Age*	23.37 ± 4.6
OCI-R score*	16.43 ± 10.37
	Median = 15, Range: 1–53**
DASS-21 depression scale score*	3.0 ± 2.3
DASS-21 anxiety scale score*	2.4 ± 2.5

*Mean ± SD format. **From the possible score range of 0–72, 21 being the clinical cutoff for OCD.

### Assessment of Personality Traits

#### Obsessive-Compulsive Tendencies

Obsessive-compulsive symptoms were measured with the obsessive-compulsive inventory-revised (OCI-R; Foa et al., 2002) that has been shown to have good validity, test-retest reliability and internal consistency in both clinical and non-clinical samples (Foa et al., 2002).

#### Depression and Anxiety Tendencies

Anxiety and depression were measured with the 21-item DASS (DASS-21; Lovibond and Lovibond, 1995a,b; Henry and Crawford, 2005) that possesses adequate convergent and discriminant validity (Henry and Crawford, 2005; Osman et al., 2012; Sinclair et al., 2012). The DASS-21 depression and anxiety scale scores, which are each based on seven items, were taken as measures of depression and anxiety tendencies.

### Experiments and Tasks

Detailed instructions were provided and color blindness was tested^1^ (Ishihara, 1917) before the experiments began. The testing session began with the supra-second timing and control tasks (Task 4, Task 3, Task 1, Task 2, see below), followed by the sub-second timing and control tasks (Task 5, Task 6, Task 7, see below), and ended with the DASS and the OCI-R questionnaires and a full debriefing interview about strategies, difficulties, and performance estimation. Breaks were allowed between tasks and between trials, as the participants controlled the beginning of each trial by pressing a button when ready. The order of the experimental tasks was kept the same for all participants, as we wanted any priming, learning or fatigue effects to be comparable across participants per task. The experiments and personality assessments were carried out in a dimly lit lab testing room on a PC (~55 cm distance, 1024 × 768 resolution, 75 Hz refresh rate), altogether lasting approximately 1 h. Stimuli were presented via the Cogent MATLAB toolbox^2^ (developed by the Cogent 2000 team at the FIL and the ICN and Cogent Graphics developed by John Romaya at the LON at the Wellcome Department of Imaging Neuroscience).

### Tasks

#### Task 1: Temporal Discrimination of Supra-Second Intervals (~9 s), Fixed Criterion

**Figure 1:**
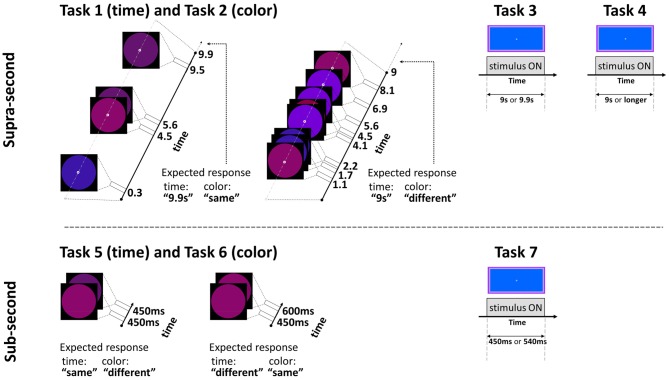
**Experimental paradigm tasks: timeline and stimuli of representative trials.** Top: supra-second tasks: fixed criterion discrimination (Tasks 1 and 3) or open-ended timing task (Task 4), and color control discrimination task (Task 2). Bottom: sub-second tasks: fixed criterion timing (Tasks 5 and 7) or color control task (Task 6). Note that same paradigm was used for timing or color perception with a change of task (Tasks 1 and 2, and Tasks 5 and 6, see Experimental Design): the temporal tasks required attending the time while ignoring the colored circles, the color tasks required attending the colors while ignoring the time; the expected responses for these experimental trials are indicated next to them. Further details are available in Experimental Design.

This fixed criterion task (Gilaie-Dotan et al., 2011, 2014) required participants to discriminate between two visually presented and *a priori* known duration intervals of 9 s and 9.9 s in a two alternative forced choice (2AFC) manner. Critically, the same paradigm also served for the non-temporal control experiment with a simple change of task (see Task 2 below and Figure 1). The intervals were represented by a small white empty circle (diameter of 0.42° of visual angle) appearing at fixation on a black background. During the interval, while the white empty circle was presented, bigger colored “distracting” circles (diameter of 6.24° of visual angle) in red, pink, purple, and blue briefly appeared (each for 200 ms) in an asynchronous fashion (SOA between 300–5475 ms, mean 1373.25 ms), giving the impression of flashing/flickering circles (see Figure 1; Coull et al., 2004). The colors’ order and the number of flickering colored circles varied across trials (3–9 circles per trial, mean of 6.25; see Figure 1).

Participants were instructed to estimate whether the small white empty circle appeared for 9 s or almost 10 s, and to respond promptly after stimulus offset while ignoring the flashing colored circles. They were told that the order of the intervals would be random. They were not instructed to use any specific method but were asked to refrain from using any motor, sensory or verbal aid (i.e., no tapping, speaking etc.). No feedback was provided. After four practice trials, participants completed 36 trials run in three different 12-trial blocks (each block consisting of six 9 s trials). To reduce expectancy, the blocks differed in the order of the flashing colored circles and their trial order, and each participant underwent all three versions. The order of versions was counterbalanced across participants.

#### Task 2: Non-Temporal Control Task for Supra-Second Intervals (~9 s), Fixed Criterion

To control for non-temporal factors such as sustained attention, here participants performed a *color* discrimination task while the physical stimuli and presentation paradigm were as described above for Task 1 (similar to previous studies, Gilaie-Dotan et al., 2011, 2014; see also Coull et al., 2004). Thus, participants were asked to attend to the color of the flashing circles (see above and Figure 1) appearing throughout the trial and judge in a 2AFC manner whether the color of the last appearing circle in a trial was identical to or different than the color of the preceding circle. They were also told that the number of flashing circles varied across trials and the circles’ presentation rate was asynchronous so the appearance of the last appearing circles could not be predicted in advance.

Importantly, the decision in the temporal task (Task 1) and in this non-temporal color task could only be reached at the end of the trial, and we also made an effort to equate the difficulty of the two tasks, based on prior results with these paradigms for 12–13 s intervals (Gilaie-Dotan et al., 2011, 2014).

#### Task 3: Temporal Discrimination of Supra-Second Intervals (~9 s) without Distractors, Fixed Criterion

In this fixed criterion task we measured ~9 s temporal estimation ability when no distractors (such as the flashing colored circles in Tasks 1 and 2) intervened, in a similar fashion to that employed in earlier studies (Brown et al., 1995; Gilaie-Dotan et al., 2011). As in Task 1, participants were required to discriminate whether the duration of a visually presented blue rectangle (10.9_width_ × 5.2_height_ visual degrees) lasted for 9 s or longer (9.9 s) in a 2AFC manner (see Figure 1). They were instructed that the blue rectangle will be presented for 9 s in half of the trials and for almost 10 s in the other half of the trials, and that the presentation order is random. Participants started with a short practice of 3–4 trials, and then performed 10 experimental trials.

#### Task 4: Temporal Sensitivity to Supra-Second Intervals (~9 s), Open Ended

**Figure 2:**
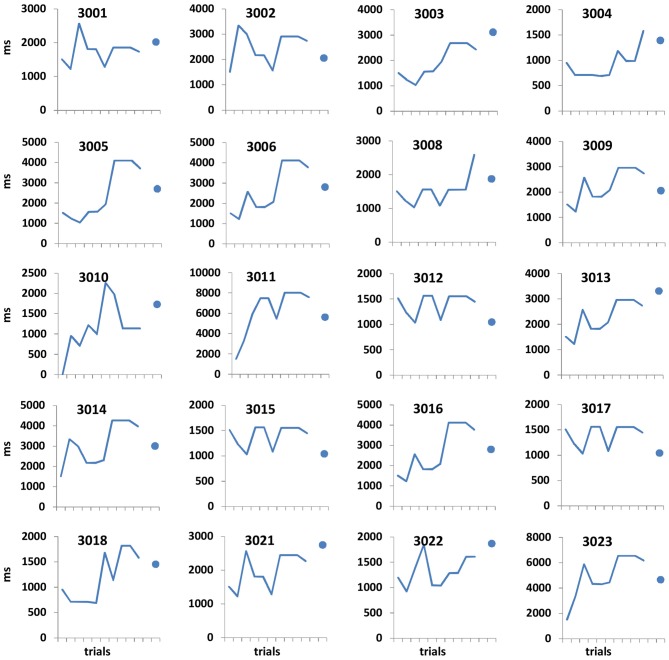
**Open-ended supra-second sensitivity estimation profiles.** Time perception sensitivity to 9 s intervals as estimated in Task 4 across 10 trials for individual participants (see “Materials and Methods” Section). Profiles of 20 arbitrarily chosen participants are presented (participant no. appears at the top of each panel). Each panel displays the approximated sensitivity in ms (y axes, note the different individual scales) at the end of each trial (x axis). The sensitivity indicates the approximated minimum Δ duration difference (in ms) that would allow the participant to distinguish between 9 s and 9 + Δs at 75% accuracy. The approximated sensitivity at the end of each trial is estimated by the efficient adaptive Bayesian QUEST algorithm (Watson and Pelli, 1983) and thus is dependent upon prior performance including that of the current trial. The final estimated sensitivity is indicated by the blue dot; note that this estimated sensitivity is also dependent upon performance on the last trial.

We conducted an additional experiment to yield a finer psychometric measure of individuals’ ~9 s intervals discrimination sensitivity. This was done by applying a Bayesian paradigm that efficiently estimates perceptual thresholds (QUEST; Watson and Pelli, 1983). Using an adaptive staircase method, we estimated the minimum duration difference enabling an individual to discriminate a 9 s interval from a longer one (i.e., the noticeable duration difference for 9 s) at 75% accuracy. In trials longer than 9 s, the duration increment (from the 9 s pedestal) was estimated based on the participant’s previous responses until that trial. Participants were asked to judge whether the appearance of a rectangle (as that described in Task 3, Brown et al., 1995) lasted for 9 s or longer, and they were informed that trials longer than 9 s could vary and be of unpredictable durations (see Figures 1, 2).

Participants underwent short training of four trials, after which they performed the 10 trial adaptive staircase procedure. Trials of 9 s were interspersed randomly among longer duration trials. The temporal perceptual threshold was defined as the estimated duration increment (Δ) that allowed each participant to discriminate 9 s and 9 + Δs at the predetermined accuracy level.

#### Task 5: Temporal Comparison of Sub-Second Intervals (450–600 ms), Fixed Criterion

In this task the participants were required to discriminate between sub-second temporal intervals (450–600 ms) in an experimental paradigm that matched the supra-second duration discrimination task (Task 1) in stimuli, setup, and employed a similar task. Each trial presented two consecutive stimulus intervals (colored circles either of the same or of (slightly) different colors, see Figure 1 and Task 6 for details) that appeared for identical or different durations. The participants were instructed that the first circle interval would be presented for 450 s in all trials, and the second interval would be presented for either the same or for longer duration. They were instructed to decide whether the second interval was of the same or of longer duration than the first interval in a 2AFC manner while ignoring the colors of the stimuli. The first interval always lasted 450 ms and the second lasted either 450 ms (same duration) or 600 ms (longer duration). No feedback was given. After a four-trial practice, the task was run in three blocks, each consisting of 12 trials (altogether 36 trials per participant), and the block order was counterbalanced across participants. In each block six trials were of “same duration” (half of these were “same color” and half of these were “different color”) and six trials were of “different duration” (half were of “same color” and half were of “different color”).

#### Task 6: Non-Temporal Control Task for Sub-Second Intervals (450–600 ms), Fixed Criterion

In this task, the physical stimuli and presentation paradigm were identical to those of the sub-second temporal task (Task 5) with only a change of task requiring to discriminate colors. Participants were asked to attend to the color of the circle stimuli that appeared and judge whether the color/shade of the second circle was identical to or different than the color of the first circle in a 2AFC manner (see Figure 1). They were informed that the color or shade differences might be small. To prevent ceiling performance effects in this task, the colors of the circles in the “different color” trials were very similar (yet different) shades of purple [RBG values = (105, 10, 126), (98, 19, 128), or (109, 0, 145)].

#### Task 7: Temporal Estimation of Sub-Second Intervals (450–540 ms), Fixed Criterion

The temporal discrimination ability for sub-second intervals was estimated using the same procedure described above in Task 3, but now for discriminating between intervals of sub-second shorter durations (450 and 540 ms—a 20% difference, see Figure 1). Specifically, they were required to discriminate whether the visual presentation of the blue rectangle (as described in Task 3) lasted for shorter (450 ms) or slightly longer (540 ms) duration in a 2AFC manner (see Figure 1). They were instructed that the presentation would be brief, similar to that of Tasks 5 and 6, and that in half of the trials the presentation will be of the shorter interval, and that the presentation order is random. Participants started with a short practice of four trials, and then performed 10 experimental trials.

### Statistical Analysis

For *t*-tests and *z* tests we only report here 2-tailed *p*-values, and for correlation analyses we only report here non-directional *p*-values. Alpha level of 0.05 was standardly used. When correlating OCI-R values with performance in each of the seven tasks, corrected alpha level due to multiple comparisons (*n* = 7) was set to 0.0071. For testing the robustness of the correlation between OCI-R scores and supra-second timing sensitivity (measured in Task 4), we used the robustfit MATLAB^3^ function.

## Results

OC symptoms in our sample, as measured in our study by the OCI-R scores (See Table 1 for details) covered a wide range of OC severity. Notably, according to published criteria for the OCI-R (Foa et al., 2002), 16 individuals (26.7%) were above the clinical OCD cutoff score of 21.

Performance details [accuracy, response times (RTs) and sensitivity measures (for Task 4)] are provided in Table 2. Accuracy in the supra-second and sub-second timing tasks was comparable across the fixed criterion tasks (around 75%) and did not reach ceiling or floor levels (see Table 2 for Tasks 1, 3, 5 and 7). Noticeable performance variance across participants was observed in the open-ended time perception sensitivity task (see Task 4 in Table 2 and Figure 2).

**Table 2:** **Performance details by task**.

Task	Accuracy* (mean ± SD [range], in %)	Response timesno. (mean ± SD, in ms)
1	75 ± 8 [58–94]	982 ± 308
2	90 ± 8 [56–100]	1355 ± 356
3	77 ± 12 [50–100]	983 ± 359
4*	2285 ± 1042 [816–5612]	
5	76 ± 9 [56–97]	1061 ± 308
6	82 ± 7 [58–94]	706 ± 233
7	76 ± 10 [55–95]	1007 ± 346

*Task 4 performance represents the estimated noticeable duration difference (in ms) required to distinguish 9 s from longer intervals.

**Figure 3:**
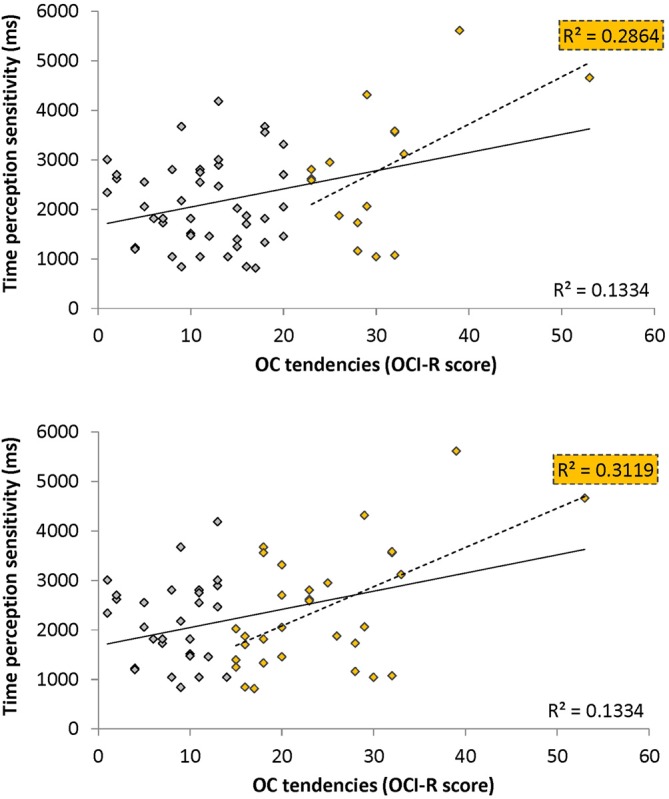
**Correlation between obsessive-compulsive (OC) tendencies OC inventory-revised (OCI-R) scores and supra-second (~9 s) time perception sensitivity (Task 4).** Supra-second (~9 s) timing sensitivity (Task 4) is significantly correlated with OC tendencies (OCI-R scores; Foa et al., 2002) such that higher OC tendencies are associated with worse sensitivity to supra-second intervals. The sensitivity (y axis in ms) indicates the Δ duration required for discriminating 9 s from 9 + Δs intervals (see “Materials and Methods” Section); the OC tendencies are presented on the x axis. As can be seen in yellow, this correlation is driven by individuals with higher OC tendencies (top panel: in yellow individuals with OCI-R score at or above 21 which is the clinical cutoff score (*n* = 16); bottom panel: in yellow individuals with OCI-R at and above the median score of 15 (*n* = 31); at the top right corner of each panel is the squared correlation coefficient (R^2^) of the high OCI-R scoring individuals indicated in that panel). The squared correlation coefficients (R^2^) of the whole cohort (*n* = 60) appears at the bottom right of each panel. Table 5 provides further statistical analysis for the whole cohort (*n* = 60) and the subgroup with OCI-R scores at or above the median score (yellow in bottom panel); similar significant results are obtained for the subgroup with OCI-R scores at or above the clinical cutoff score (*n* = 16, *r* = 0.535, *t*_(14)_ = 2.369, *p* = 0.0327).

To test our main hypothesis that OC tendencies would be associated with supra-second timing, we correlated OCI-R scores with supra-second timing performances (accuracy in Tasks 1, 3, and sensitivity in Task 4, see Table 3). In contrast to our hypothesis, supra-second timing performance in the fixed criterion tasks was not correlated with OC tendencies (Task 1: *r* = 0.04, *t*_(58)_ = 0.31, *p* = 0.757; Task 3: *r* = −0.01, *t*_(58)_ = −0.08, *p* = 0.936; see also Table 3). However, timing sensitivity as measured by the open-ended task (Task 4) was significantly correlated with OC tendencies (*r* = 0.37, *t*_(58)_ = 2.99, *p* = 0.004, see Table 3 and Figure 3), even when correcting for multiple comparisons or testing for its robustness (*p* < 0.05; see “Materials and Methods” Section). Furthermore, this correlation was significantly or marginally significantly different from the two other correlations between OC tendencies and the fixed criterion supra-second tasks (see Table 3 bottom row). To examine whether high OCI-R scoring individuals are driving this correlation, we examined whether the correlation within the high-scoring individuals (OCI-R score at or above the clinical cutoff score of 21, *n* = 16) was significant, and whether it was significantly different from the correlation within the low-scoring individuals (OCI-R score of 9 or below, *n* = 16). As can be seen in Table 4, we found a significant correlation between OCI-R score and timing sensitivity (Task 4) only in the high-scoring group (OCI-R score > = 21, *n* = 16; *r* = 0.535, *t*_(14)_ = 2.37, *p* = 0.0327), but not in the low-scoring group (OCI-R score < = 9, *n* = 16; *r* = −0.2138, *t*_(14)_ = −0.819, *p* = 0.426), and these correlations were significantly different from each other (*z* = 2.08, *p* = 0.0375). Table 4 further shows that these groups were significantly different in many personality tendencies yet did not differ in accuracy or RTs for any of the study tasks. We further split our entire cohort into the higher OC tendencies half (at and above the OCI-R median score of 15 in our sample, *n* = 31), and the lower OC tendencies half (below the median score, *n* = 29), and again found a significant correlation only in the higher OC tendency half (*r* = 0.56, *t*_(29)_ = 3.62, *p* = 0.0011, *n* = 31) but not in the lower OC scoring half (*r* = 0.04, *t*_(27)_ = 0.20, *p* = 0.84, *n* = 29; see Figure 3 and Table 5), with these correlations being significantly different from each other (*z* = 2.18, *p* = 0.0293). These results indicate that the relationship between OC tendencies and timing sensitivity in the open-ended task was predominantly driven by people with high OC tendencies.

**Table 3:** **Correlations between supra-second tasks and OC tendencies**.

	Fixed criterion temporal discrimination accuracy (Task 1, with distractors)	Control color task: Fixed criterion discrimination (Task 2)	Fixed criterion temporal discrimination accuracy (Task 3, no distractors)	Open ended temporal sensitivity (Task 4, no distractors)
**OC tendency (OCI-R overall score)**	*r* = 0.041, *t*_(58)_ = 0.313, *p* = 0.757	*r* = −0.105, *t*_(58)_ = −0.804, *p* = 0.253	*r* = −0.011, *t*_(58)_ = −0.084, *p* = 0.936	*r*** = 0.365**, ***t*_(58)_** = 2.986, *p* = 0.004*
**Correlation differences significance (vs. Task 4)**	*z = 1.83,* *p = 0.0673*	*z*** = 2.85,** *p*** = 0.004**	*z*** = 2.1,** *p*** = 0.036**	**—**

*Top row: significant correlations are shown in bold font. For further information see the “Results” Section. Bottom row: significance of correlation differences, comparing each correlation presented in the top row to the one between OCI-R overall score and Task 4 open ended temporal sensitivity. Significant results are shown in bold font, results tending to significance (p < 0.1) are shown in italics*.*Survives correction for multiple comparisons.

**Table 4:** **Comparison between lowest-scoring and highest-scoring OCI-R individuals, across personality and experimental performance**.

		Lowest scoring	Highest scoring	Difference (*t*_(30)_, *p*)
	*n*	16	16	
	**Task 4 correlation with OCI-R score**	*r* = −0.2139, *t*_(14)_ = −0.819, *p* = 0.43	*r* = 0.535, *t*_(14)_ = 2.37, *p* = 0.033	***z* = 2.08,** ***p* = 0.0375**
**Personality tendencies**	**OCI-R scores (mean ± SD [range])**	5.4 ± 2.9 [1–9]	30.3 ± 7.4 [21–53]	**2.42, 0.0217**
	**DASS total**	7.8 ± 6.2	18.2 ± 8.5	**3.96, <0.001**
	**DASS depression**	2.1 ± 2.4	4.1 ± 2.5	**2.26, 0.03**
	**DASS anxiety**	1.6 ± 1.7	4.5 ± 3.2	**3.34, 0.002**
**Experimental performance**	**Task 1 accuracy**	73.6 ± 7.9	75.9 ± 6.6	0.88, 0.39
	**Task 1 RT**	976.5 ± 371	984.8 ± 278.9	0.07, 0.94
	**Task 2 accuracy**	92.4 ± 5.2	88.9 ± 7.9	1.5, 0.15
	**Task 2 RT**	1289.0 ± 352.7	1376.4 ± 329.2	0.72, 0.47
	**Task 3 accuracy**	76.9 ± 12.5	80.0 ± 13.2	0.69, 0.50
	**Task 3 RT**	1051.6 ± 408.2	949.0 ± 318.1	0.79, 0.43
	**Task 4 sensitivity (estimated duration difference)**	2098.4 ± 786.2	2796.1 ± 1325.1	*1.81, 0.08*
	**Task 5 accuracy**	76.6 ± 8.5	77.8 ± 9.4	0.38, 0.70
	**Task 5 RT**	1038.3 ± 357.0	1030.9 ± 266.0	0.07, 0.95
	**Task 6 accuracy**	81.4 ± 6.3	80.7 ± 5.7	0.33, 0.75
	**Task 6 RT**	698.1 ± 193.7	736.0 ± 232.8	0.5, 0.62
	**Task 7 accuracy**	80.1 ± 9.2	74.1 ± 12.6	1.55, 0.13
	**Task 7 RT**	951.2 ± 256.6	930.5 ± 319.4	0.20, 0.84

*Accuracy rates in %, RTs in ms, estimated duration difference in ms*.

OCD commonly co-occurs with depression and anxiety (American Psychiatric Association, 2013), and indeed this was the case in our sample (OCI-R to depression scores: *r* = 0.40, *t*_(58)_ = 3.34, *p* = 0.001; OCI-R to anxiety scores: *r* = 0.59, *t*_(58)_ = 5.57, *p* < 0.0001). Therefore, we examined whether the significant correlation between open-ended supra-second time sensitivity (Task 4) and OC tendencies could be explained by these factors. As can be seen in Table 5, partial correlation analysis revealed that the correlation was still significant when controlling separately for anxiety or depression levels as assessed by the DASS-21, and nearly significant when controlling for the two factors together. Importantly, in the higher OC tendency group (either taken as the participants scoring at or above the median OCI-R score (*n* = 31) or taken as the participants scoring above the clinical cutoff (*n* = 16) the correlation remained significant after controlling for all these symptoms separately or together (see further details in Table 5).

**Table 5:** **Correlations between the supra-second open-ended temporal sensitivity task (Task 4) and OC tendencies**.

Overall OC tendency (OCI-R score)	*n*	Open ended temporal sensitivity (Task 4, no distractors)
**OC tendency (overall)**	60	***r* = 0.365, *t*_(58)_** = 2.986, *p* = 0.004
**Controlled for depression**	60	***r* = 0.304, *t*_(58)_** = 2.43, *p* = 0.019
**Controlled for anxiety**	60	***r* = 0.267, *t*_(58)_** = 2.11, *p* = 0.039
**Controlled for depression**	60	*r = 0.251, t_(58)_ = 1.97, p = 0.056*
**and anxiety together**		
**Score at/above median (> = 15)**	31	***r* = 0.558, *t*_(29)_** = 3.621, *p* = 0.0011
**Controlled for depression**	31	***r* = 0.4911, *t*_(29)_** = 3.036, *p* = 0.0059
**Controlled for anxiety**	31	***r* = 0.4177, *t*_(29)_** = 2.476, *p* = 0.0216
**Controlled for depression and anxiety together**	31	***r* = 0.399, *t*_(29)_** = 2.35, *p* = 0.032
**Score below median (<15)**	29	*r* = 0.038, *t*_(27)_ = 0.198, *p* = 0.8445

*Significant correlations are shown in bold font, correlations tending to significance (p < 0.1) are shown in italics. For further information see the “Results” Section and Figure 3*.

Supra-second (typically above 3 s) and sub-second timing (typically up to 1 s) rely on different perceptual (e.g., Elbert et al., 1991; Poppel, 1997; Lewis and Miall, 2003a,b; Ulbrich et al., 2007; Morillon et al., 2009; Gilaie-Dotan et al., 2011) and different neural processes (e.g., Elbert et al., 1991; Poppel, 1997; Lewis and Miall, 2003a,b; Ulbrich et al., 2007; Morillon et al., 2009; Gilaie-Dotan et al., 2011). More specifically, timing processes of up to 1 s (“sub-second”) are assumed to rely on motor-based and also sensory-based mechanisms and thus termed “automatic timing”, whereas timing of intervals longer than 3 s (“supra-second”) are assumed to rely in addition on more cognitive mechanisms allowing the perception of the accumulating interval, thus termed “cognitive timing”. This dissociation between supra-second and sub-second timing is also evident in our data (correlation between Task 1 and Task 5: *r* = 0.12, *t*_(58)_ = 0.94, *p* = 0.352; between Task 3 and Task 7: *r* = 0.12, *t*_(58)_ = 0.89, *p* = 0.38, see Figure 1). Therefore, we also examined the associations between sub-second fixed-criterion timing performance and OC tendencies. As can be seen in Table 6, only Task 7 performance was correlated with OCI-R scores, but it did not survive multiple-comparisons correction or controlling for depression or anxiety.

**Table 6:** **Correlations between sub-second tasks and OC tendencies**.

	Time discrimination (Task 5)	Color discrimination (Task 6)	Time discrimination without distractors (Task 7)
**OC tendency (OCI-R overall score)**	*r* = 0.184,	*r* = −0.071,	***r* = −0.254,**
****	*t*_(58)_ = 1.426,	*t*_(58)_ = −0.543,	***t*_(58)_** = −2.0,
****	*p* = 0.160	*p* = 0.589	***p* = 0.05**
**Correlation differences significance (vs. Task 4)**	*z* = 1.05,	***z* = 2.42,**	***z* = 3.43,**
****	*p* = 0.2937	***p* = 0.0155**	***p* = 0.0006**

*Presentation conventions are identical to Table 3. Top row: no results survived correction for multiple comparisons, depression or anxiety. Bottom row: significance of correlation differences, comparing each correlation presented in the top row to the one between OCI-R overall score and Task 4 open ended temporal sensitivity (see also Table 3)*.

RTs or speed-accuracy measures (RT/accuracy) were not significantly associated with OCI-R scores in any of the tasks (all r’s < 0.15, |*t*_(58)_|’s < 1.16, p’s > 0.25). Performances on the non-temporal color control tasks were, as expected, uncorrelated with timing performance (supra-second Task 1 with Task 2: *r* = 0.11, *t*_(58)_ = 0.83, *p* = 0.41; sub-second Task 5 with Task 6: *r* = 0.08, *t*_(58)_ = 0.57, *p* = 0.58), consistent with previous findings (Coull et al., 2004; Gilaie-Dotan et al., 2011). Importantly, and as expected, performance on the control color tasks was not correlated with OC tendencies (see Tables 3, 6).

## Discussion

Following recent findings suggesting that OCD is related to attenuated access to internal states in various domains, including interoception (Lazarov et al., 2010, 2012a,b), the present study examined whether OC tendencies are associated with reduced timing abilities, especially in the supra-second range. We measured OC tendencies and employed a series of tasks to measure supra-second timing and control tasks in a group of 60 university students that spanned the typical adult OC symptom range. We found that open-ended supra-second timing performance was correlated with OC tendencies, as predicted, whereas all the other tasks were not. This correlation was especially evident in individuals with higher OC tendencies, where OC tendencies were negatively associated with supra-second timing sensitivity. However, there are a number of factors that currently limit the generalization and conclusions that can be drawn from our study (for more details, see “Study Limitations” Section below).

### Relationship Between OC Tendencies and Supra-Second Timing

We found that open-ended supra-second timing sensitivity, but not the fixed-criterion tasks, were associated with OC tendencies. There are at least two explanations for this finding. First, different levels of uncertainty may be associated with these different tasks. The fixed criterion timing tasks presented one of two intervals of predetermined durations (9 or 9.9 s) in each trial. In contrast, the open-ended task presented interval durations of 9 s or longer and participants were told that the longer intervals could be of any duration, a situation that may have involved a higher degree of uncertainty than the fixed criterion tasks. Second, fixed criterion tasks might have allowed for indirect contextual feedback, which was not available in the open-ended task. For example, some participants reported that in the fixed criterion tasks they relied on a counting strategy and were able at times to infer that they counted too fast (e.g., getting up to 11) or too slow (e.g., getting only up to 8) and thus were able to improve their performance along the experiment. This was not the case in the open-ended task, where they felt greater uncertainty regarding both the duration of the interval and their own performance.

The possibility that uncertainty and lack of feedback contributed to the relationship between the open-ended timing and OC tendencies is in line with the SPIS hypothesis. As discussed above, the SPIS model suggests that people with OC tendencies rely on proxies to compensate for their attenuated access to internal states, including interoceptive sensations (Liberman and Dar, 2009; Lazarov et al., 2010, 2012a). In our experiment, indirect feedback that was available in the fixed criterion tasks (as only two fixed interval durations were employed, so one could have used strategies to distinguish between them) might have served as a timing proxy for the high OC participants. Moreover, a recent study examining rodent timing suggests that rhythmical behavior in rodents could be linked to human repetitive motor habits that are commonly observed in OCD (Gu et al., 2011). These repetitive motor habits in OCD often include rituals or compulsions that may temporarily reduce anxiety (American Psychiatric Association, 2013) like double checking locks, counting, tapping, or other repetitive behaviors. That study suggests that in OCD such repetitive behaviors might have a tendency to be synchronized with specific temporal regularities in the environment that are normally inhibited (Gu et al., 2011). Therefore, the mechanisms supporting these repetitive motor habits in OCD, which could be relying on broader neural circuits, might be able to provide timing proxies in OCD even when no motor action is being performed. In our study, the indirect feedback available in the fixed criterion tasks (but not in the open-ended task), might have been mentally associated with a repetitive action to serve as temporal proxy for OC tending individuals. Further research is needed to substantiate the hypothesis that providing feedback or other proxies for timing under uncertainty might have significant effects on the timing performance of people with high OC tendencies.

Our findings are consistent with earlier studies that show that interoception as in awareness to internal states and bodily sensations is positively correlated with timing in the supra-second range. For example, higher heartbeat perception correlates with higher supra-second reproduction accuracy (Meissner and Wittmann, 2011), and HRV is associated with accurate time perception. Two studies that investigated how timing perception relates to multiple physiological factors including HRV, found that higher vagal heart control is linked to higher accuracy in timing perception both in sub-second (Cellini et al., 2015) and in supra-second timing (Pollatos et al., 2014b). In addition, interoceptive attention (to body and bodily reactions) was shown to significantly affect retrospective time estimation of emotional film clips relative to exteroceptive attention (Pollatos et al., 2014a), where amusement leads to quicker passage of time and fear to slowing down of time. In fact studies from as early as the 1920’s and 1930’s show that body temperature influences supra-second time estimation (François, 1927; Hoagland, 1933, 1935; Wearden and Penton-Voak, 1995; Wearden, 2005), and these—it has been suggested—might have led to the development of internal clock theory (e.g., Treisman, 1963; Gibbon et al., 1984; and see review in Wearden, 2005). In these very early studies, higher body temperature led to speeding up of the perceived time whereas lower body temperature led to the slowing down of the perceived time (François, 1927; Hoagland, 1933, 1935; Wearden and Penton-Voak, 1995; Wearden, 2005). The internal clock model proposes that timing performance relies on memory storage of a reference interval, and when compared with a pacemaker that follows a characteristic frequency, and depending on the sensory arousal state, timing performance is determined (Treisman, 1963; Treisman et al., 1990, 1994; Wearden, 2005; Allman et al., 2014). The uncertainty and persistent feeling of doubt in OC tendency that leads to intrusive thoughts and sometimes actions, might each interfere with any of the internal clock components. So for example, as proposed by Treisman et al. (1990, 1994) at later stages, arousal, emotional states or sensory inputs might perturb the characteristic frequency of the pacemaker and thus cause over or under estimation of time. Intrusive thoughts might interfere with the ability to sustain the reference interval in memory. Emotional states as heightened anxiety or stress might induce overall noise to multiple components of the internal clock, reducing the ability to achieve veridical timing. The striatal beat frequency model (Buhusi and Meck, 2005) suggests how the fronto-striatal neural machinery might actually implement the internal clock model, with the striatum acting as the comparator, and the cortical oscillatory neurons as the pacemaker. According to the striatal beat frequency model, the fronto-striatal activity that is modulated in OC tending individuals (Kathmann et al., 2005; Maltby et al., 2005; van den Heuvel et al., 2005; Remijnse et al., 2006; Simon et al., 2010; Freyer et al., 2011) might adversely affect their timing performance. However, OC symptomatology is diverse (spanning a manifold of typical obsessions and compulsions), interoception awareness encompasses a variety of bodily sensations (e.g., temperature, heartbeat, visceral, muscular, hunger, and pain), and supra-second timing might rely on multiple neuropsychological mechanisms for different timing tasks (e.g., retrospective vs. prospective timing, estimation vs. reproduction). Therefore, whether these relationships between OC tendencies, supra-second timing, and interoceptive awareness hold under the different manifestations, how robust they are, which components of the internal clock might be affected in OC timing, and their causal or epiphenomenal nature are yet to be substantiated.

Interestingly, multiple studies and theories suggest that interoceptive awareness, OC tendencies and supra-second timing are all linked to the insula. For example, insular activity and neuroanatomy are associated with and suggested to be the basis for interoception and bodily awareness (Craig, 2003, 2009; Critchley et al., 2004; Wittmann et al., 2010). Furthermore, following recent views that suggest that supra-second timing involves and might not be dissociated from emotional and interoceptive states (see Wittmann, 2009), the insula has been suggested as a neurophysiological mechanism for the encoding of duration. This is supported by both activation and neuroanatomical studies (Critchley et al., 2004; Craig, 2009; Wittmann et al., 2010; Gilaie-Dotan et al., 2011; Wittmann, 2013). Consistent with this idea, studies investigating OCD have reported increased insular activity in patients relative to controls when viewing OC-relevant disgusting pictures (Phillips et al., 2000; Shapira et al., 2003), and also altered resting state functional connectivity between the anterior insula and the default mode network in OCD patients relative to controls (Stern et al., 2012).

### Study Limitations

Several limitations of the present study should be noted and perhaps addressed in future research. First, our study included students rather than clinically diagnosed individuals with OCD. Although the use of a non-clinical sample is common and has been shown to be useful (Gibbs, 1996; Nedeljkovic and Kyrios, 2007; Abramowitz et al., 2014), it would still be beneficial to test this hypothesis in the clinical OCD population. A second limitation is the limited number of participants with high OC tendency scores (only six with OCI-R score >30). Testing a cohort with larger number of high OC scoring individuals is important for strengthening the results reported in our study. A third limitation is that we did not employ an open-ended sub-second timing task that would have allowed comparing sub-second open-ended timing to OC tendencies. Therefore, we cannot rule out a possibility that open-ended timing, whether sub-second or supra-second, could be associated with OC tendencies. Furthermore, we examined timing with a specific set of timing tasks (interval estimation and discrimination) from a wide range of tasks employed in timing research (e.g., temporal bisection, reproduction, etc.) which might each tap into different mechanisms. Another limitation is that our study was not *a priori* designed to examine time estimation in OC tendencies under different uncertainty levels. In addition, the non-temporal control tasks (color tasks, Tasks 2 and 6) did not include an open-ended task that may have been useful in controlling for possible uncertainty. Therefore, we cannot rule out that factors not specific to timing, as uncertainty levels, can explain OC tendencies’ association with the open-ended time sensitivity performance, rather than timing *per se*. The unbalanced gender distribution in our study stems from the uneven gender distribution amongst psychology students, as has been the case in our previous studies with non-clinical population (e.g., Lazarov et al., 2010, 2012a,b). Nevertheless as gender distribution is rather even in OCD, this factor is unlikely to limit the generalization of the results to other non-clinical OC tending individuals.

## Conclusions

The present study suggests that attenuation of access to internals states, which has been proposed as a central phenomenon of OCD, might be relevant to the domain of time perception. As timing perception plays a multiplicity of roles in everyday lives, further investigations into its role in OCD might prove useful in furthering our understanding of this complex and difficult disorder. Investigating subclinical populations as in this study not only provides access to a large number of individuals with OC tendencies, but may also provide insights about the spectra and magnitude of the OC manifestations as well as subtler insights into the phenomenology of the disorder.

## Author Contributions

SG-D and RD conceived the study, SG-D, HA, and RD designed the study. HA collected the data, SG-D and HA analyzed the data, SG-D, HA, and RD wrote the article.

## Conflict of Interest Statement

The authors declare that the research was conducted in the absence of any commercial or financial relationships that could be construed as a potential conflict of interest.

## Footnotes

^1^http://colorvisiontesting.com/ishihara.htm, as appeared during 2013

^2^http://www.vislab.ucl.ac.uk/cogent_2000.php

^3^www.mathworks.com
